# Modified fish diet shifted serum metabolome and alleviated chronic anemia in bottlenose dolphins (*Tursiops truncatus*): Potential role of odd-chain saturated fatty acids

**DOI:** 10.1371/journal.pone.0230769

**Published:** 2020-04-07

**Authors:** Stephanie Venn-Watson, Mark Baird, Brittany Novick, Celeste Parry, Eric D. Jensen

**Affiliations:** 1 Translational Medicine and Research Program, National Marine Mammal Foundation, San Diego, California, United States of America; 2 U.S. Navy Marine Mammal Program, Naval Information Warfare Center Pacific, San Diego, California, United States of America; University of Leeds, UNITED KINGDOM

## Abstract

Bottlenose dolphins (*Tursiops truncatus*) are long-lived mammals that can develop chronic aging-associated conditions similar to humans, including metabolic syndrome. Initial studies suggest that these conditions may be attenuated in dolphins using a modified fish diet. Serum metabolomics, fatty acid panels, and blood-based health indices were compared between 20 dolphins on a modified, 50% wild-type diet (50% mullet, 25% capelin, and 25% squid and/or herring) and 10 dolphins on a baseline diet (75% capelin and 25% squid and/or herring). Blood samples were collected at Months 0, 1, 3 and 6. Dolphins on the modified diet had lower insulin (7.5 ± 4.0 and 14.8 ± 14.0 μIU/ml, *P* = 0.039), lower cholesterol (160 ± 26 and 186 ± 24 mg/dl, *P* = 0.015) and higher hematocrit (46 ± 3 and 44 ± 3%, *P* = 0.043) by Month 1 compared to controls. Dolphins with anemia (hemoglobin ≤ 12.5 g/dl, n = 6) or low-normal hemoglobin (12.5–13.5 g/dl, n = 3) before placed on the modified diet had normal hemoglobin concentrations (> 13.5 g/dl) by Month 3. The modified diet caused a significant shift in the metabolome, which included 664 known metabolites. Thirty prioritized metabolites at Months 1 and 3 were 100% predictive of dolphins on the modified diet. Among 25 prioritized lipids, 10 (40%) contained odd-chain saturated fatty acids (OCFAs); C15:0 was the highest-prioritized OCFA. Increased dietary intake of C15:0 (from 1.3 ± 0.4 to 4.5 ± 1.1 g/day) resulted in increased erythrocyte C15:0 concentrations (from 1.5 ± 0.3 to 5.8 ± 0.8 μg/ml, *P* < 0.0001), which independently predicted raised hemoglobin. Further, increasing age was associated with declining serum C15:0 (R^2^ = 0.14, P = 0.04). While higher circulating OCFAs have been previously associated with lower risks of cardiometabolic diseases in humans, further studies are warranted to assess potential active roles of OCFAs, including C15:0, in attenuating anemia.

## Introduction

Bottlenose dolphins (*Tursiops truncatus*) are long-lived, large-brained mammals that can develop comorbidities of aging similar to humans, including chronic inflammation, elevated cholesterol, elevated insulin, and fatty liver disease [1–3]. Obesity and lower physical activity, two key risk factors for these comorbidities in humans, do not appear to be primary drivers for development of these conditions in dolphins [4,5]. While dolphin diets are primarily limited to fish, different wild and managed dolphin populations eat different species of fish on different schedules, which, in turn, vary in nutritional content by species and season [4,5].

Our previous studies have demonstrated that changing dietary fish types for dolphins could alter their serum fatty acid profiles and that these changes may lead to improved metabolic health [5]. We initially found that, when comparing wild and US Navy dolphins, higher serum concentrations of heptadecanoic acid (C17:0), a trace odd-chain saturated fatty acid (OCFA), were predictive of lower, healthier serum insulin concentrations. Further, serum C17:0 concentrations could be effectively increased in dolphins using a modified fish diet with higher amounts of OCFAs. We tentatively concluded that increased serum C17:0 concentrations may have contributed to the observed normalization of hyperlipidemia and hyperinsulinemia among dolphins on the modified fish diet. This pilot study, however, included a small number of animals, lacked a strong control group, and had analyses limited to clinical chemistries and a fatty acid panel.

OCFAs are trace saturated fatty acids present in some types of fish, whole dairy fat, and some plants [6–8]. Consistent with our findings in dolphins, higher circulating concentrations of OCFAs in humans have been repeatedly associated with lower risks of having or developing chronic conditions, including adiposity, chronic inflammation, metabolic syndrome, type 2 diabetes, nonalcoholic fatty liver disease, chronic obstructive pulmonary disease, and pancreatic cancer [9–18]. While circulating concentrations of the most commonly measured OCFAs, pentadecanoic acid (C15:0) and heptadecanoic acid (C17:0), have traditionally been used as biomarkers of dietary dairy fat intake in humans [6], active intervention studies to evaluate the potential health benefits of OCFAs have been lacking.

To better understand the potential roles of different fish-based diets and increased dietary OCFAs on health in dolphins, a larger study was conducted with 30 dolphins (20 on a modified diet and 10 controls).This study included hematological and clinical chemistry measurements, as well as global serum metabolomics, which provided a broad, less-biased assessment of changing serum biochemicals caused by the modified diet that may impact metabolism and associated health in dolphins.

## Materials and methods

### Animal care and use

The MMP is accredited by the Association for Assessment and Accreditation of Laboratory Animal Care International and adheres to the national standards of the United States Public Health Service Policy on the Humane Care and Use of Laboratory Animals and the Animal Welfare Act. As required by the Department of Defense, the Program’s animal care and use program is routinely reviewed by the MMP’s Institutional Animal Care and Use Committee (IACUC) and the Navy Bureau of Medicine and Surgery. This study was conducted under the MMP IACUC-approved animal care and use protocol #101–2012 (BUMED NRD-801).

### Study population

The study population was from a managed population of bottlenose dolphins (*Tursiops truncatus*) living in San Diego Bay, California (32.6500°N, 117.1900°W) cared for by the U.S. Navy Marine Mammal Program (MMP). Dolphins placed on the modified diet (n = 20) and dolphins maintained on the baseline diet (n = 10) were matched by age and sex. MMP dolphins live in netted enclosures within San Diego Bay, and many dolphins have daily open ocean activity sessions. MMP dolphins are fed high-quality, frozen-thawed whole fish diets consisting of primarily capelin (*Mallotus villosus*), as well as herring (*Clupea harengus*), mackerel (*Scomber japonicus*), and/or squid (*Loligo opalescens*). Routine diets are based on kilocalories per kilogram of dolphin body weight and pre-established caloric needs that are based on targeted body weights that vary by age, sex, and activity level of each dolphin. MMP dolphins are typically fed their daily intake over three to eight meals between 08:00 and 15:00. Before 1989, most MMP dolphins originated from the Gulf of Mexico, especially Mississippi Sound. Since 1989, MMP dolphins have been born at the MMP facility in San Diego Bay. All dolphins are routinely provided daily vitamin supplements (Mazuri^®^Vita-Zu^®^ Mammal Tablet, Formula Number 5M26). No dolphins in the study were on chronic medications, including antibiotics or hormonal therapy.

### Dietary intervention study

The baseline diet was based on a typical Navy dolphin diet fed over the past decade (75% kcals capelin + 25% kcals herring/squid). The modified diet (50% kcals mullet + 25% kcals capelin + 25% kcals herring/squid) aimed to increase dietary odd-chain saturated fatty acid intake based upon the original pilot study [5]. All fish in the study were wild caught (not farmed) and stored frozen at the US Navy facility before being thawed and fed to the dolphins. To control for potential variation in nutrient content of fish based on the season that fish were caught, single-sourced lots of each fish species were fed to the dolphins during this study. Capelin (*Mallotus villosus*), which typically feed on phytoplankton and zooplankton, were relatively smaller fish primarily caught off the coast of Iceland, while mullet (*Mugil cephalus*), which primarily eat zooplankton, were relatively larger fish caught in the Gulf of Mexico. All fish lots fed to dolphins during the study were analyzed for kilocalories, water, fat, protein, carbohydrate content, and fatty acid content. Nutrient intake before and during the study was calculated for each dolphin based on individual daily diets to assess changes in nutrients ingested due to the modified diet.

To normalize the study population, all study animals (n = 30) were placed on 75% kcals capelin and 25% kcals herring/squid for 8 weeks prior to the start of the study (January through February). Twenty dolphins were then moved to the modified diet for six months (March through August). Ten dolphins, housed in the same environment, were maintained on the baseline diet to control for potential non-dietary environmental factors that may have occurred during the study. All dolphins throughout the study (those placed on the modified diet and the control group) were held to the same feeding schedule (i.e. one-third of their daily diet fed at 08:00, remaining two-thirds fed between 10:00 and 15:00, and overnight fasts from approximately 15:00 to 08:00). All dolphins remained on the population’s baseline daily vitamin supplement regimen.

### Sample collection

Previously reported sample collection protocols for MMP dolphins were used in this study [5]. Briefly, one-third of the daily diet was fed in the morning after a routine overnight fast. Two-hour postprandial, in-water, and trained blood samples were drawn (typically near 10:00 a.m.). Due to 1) the long duration and large size of our study population, 2) the desire to not interfere with dolphins’ daily activities, and 3) the need to limit the number of blood draws from each dolphin, we limited our study design to single, 2-hour post-prandial blood samples at Months 0, 1, 3, and 6. The 2-hour postprandial period was selected as the most ideal time for the purpose of our study, as it would most likely capture absorbed exogenous, dietary nutrients while still in the serum (*versus* after being metabolized and excreted and/or integrated into tissues). To evaluate longer-term (vs. short-term postprandial wave) impacts of the modified diet on fatty acid profiles, we included fatty acid measurements in erythrocyte membranes, which reflect longer-term integration of dietary fatty acids into cell membranes.

Blood was collected into BD Vacutainer serum separator tubes (5.6 ml for insulin, iron, ferritin, serum fatty acids profile, and serum chemistry), EDTA BD Vacutainer blood collection tubes (8 ml for erythrocyte fatty acid profile), and Lithium Heparin BD Vacutainer blood collection tubes (4 ml for plasma chemistry, including triglycerides). Blood tubes were centrifuged at 3000 rpm for 10 minutes within 30 to 60 minutes of collection and chilled during processing until shipment. The EDTA and lithium heparin tubes were shipped on cold packs to the reference laboratories. Remaining serum/plasma was transferred to cryovials and stored at -80°C until shipment on dry ice via overnight courier to the reference laboratories.

### Hematology and clinical chemistries

Plasma clinical chemistries were analyzed as previously described [3]. Iron, TIBC, and ferritin were analyzed at the Kansas State Veterinary Diagnostic Laboratory by colorimetric analysis on the Roche Cobas Mira (Roche Diagnostics, Indianapolis, Indiana 46250) per the manufacturer’s protocol. Plasma clinical chemistries were directly measured using the Roche Cobas 8000 system (Roche Diagnostics, Indianapolis, Indiana 46250) per the manufacturers’ protocol. Total insulin was analyzed at ARUP Laboratories by ultrafiltration/quantitative chemiluminescent immunoassay on the Siemens ADVIA Centaur Immunoassay system (Siemens Medical Solutions USA, Inc., Malvern, Pennsylvania 19355). Erythrocyte sedimentation rate (ESR) was measured through a technique correlating directly with the Westergren method using the Fisher Healthcare^™^ Dispette^™^ 2 and reservoirs pre-filled with 0.25mL of 0.9% saline (Thermo Fisher Scientific). Low hemoglobin, the standard definition of anemia, was defined in our study population as animals with hemoglobin at or below the 25^th^ percentile (< 12.5 g/dl) among all animals at baseline. Low-normal hemoglobin was defined as animals greater than the 25^th^ and equal to or lower than the 50^th^ percentile (12.6–13.5 g/dl). These values are consistent with definitions for anemia in humans (< 12–14 g/dl) [19].

### Metabolomics

#### Sample preparation

Serum samples archived at -80°C were prepared using the automated MicroLab STAR^®^ system (Hamilton Company). Proteins were precipitated with methanol under vigorous shaking for 2 min (Glen Mills GenoGrinder 2000) followed by centrifugation. The resulting extract was divided into five fractions: two for analysis by two separate reverse phase (RP)/UPLC-MS/MS methods with positive ion mode electrospray ionization (ESI), one for analysis by RP/UPLC-MS/MS with negative ion mode ESI, one for analysis by HILIC/UPLC-MS/MS with negative ion mode ESI, and one sample reserved for backup. Samples were placed briefly on a TurboVap^®^ (Zymark) to remove the organic solvent. The sample extracts were stored overnight under nitrogen before preparation for analysis.

#### Quality assurance and control

Several types of controls were analyzed in concert with the experimental samples: a pooled matrix sample generated by taking a small volume of each experimental sample (or alternatively, use of a pool of well-characterized human plasma) served as a technical replicate throughout the data set; extracted water samples served as process blanks; and a cocktail of QC standards that were carefully chosen not to interfere with the measurement of endogenous compounds were spiked into every analyzed sample, allowed instrument performance monitoring and aided chromatographic alignment.

#### Bioinformatics

The informatics system consisted of four major components, the Laboratory Information Management System (LIMS), the data extraction and peak-identification software, data processing tools for QC and compound identification, and a collection of information interpretation and visualization tools for use by data analysts. The hardware and software foundations for these informatics components were the LAN backbone, and a database server running Oracle 10.2.0.1 Enterprise Edition. Peaks were quantified using area-under-the-curve. For studies spanning multiple days, a data normalization step was performed to correct variation resulting from instrument inter-day tuning differences. Essentially, each compound was corrected in run-day blocks by registering the medians to equal one (1.00) and normalizing each data point proportionately (termed the “block correction”). For studies that did not require more than one day of analysis, no normalization is necessary, other than for purposes of data visualization. In certain instances, biochemical data may have been normalized to an additional factor (e.g., cell counts, total protein as determined by Bradford assay, osmolality, etc.) to account for differences in metabolite levels due to differences in the amount of material present in each sample. Two-way ANOVA main effects models, including study group, month, and sex, were used to determine primary drivers of differences in the metabolome.

#### Principal components analysis and hierarchical clustering

Each principal component was a linear combination of every metabolite and the principal components were uncorrelated. The number of principal components was equal to the number of observations. The first principal component was computed by determining the coefficients of the metabolites that maximized the variance of the linear combination. The second component found the coefficients that maximize the variance with the condition that the second component was orthogonal to the first. The third component was orthogonal to the first two components and so on. The total variance was defined as the sum of the variances of the predicted values of each component (the variance is the square of the standard deviation), and for each component, the proportion of the total variance was computed. Hierarchical clustering was used as an unsupervised method for clustering the data to show any large-scale differences. Complete clustering using the Euclidean distance was applied, where each sample was a vector with all metabolite values.

#### Random forest regression

Random forest, a supervised classification technique based on an ensemble of decision trees, was used to provide “importance” rank ordering of serum biochemicals that changed due to the modified diet. A random subset of the data with identifying true class information was selected to build the tree (“bootstrap sample” or “training set”), and then the remaining data, the “out-of-bag” (OOB) variables, were passed down the tree to obtain a class prediction for each sample. This process was repeated thousands of times to produce the forest. The final classification of each sample was determined by computing the class prediction frequency (“votes”) for the OOB variables over the whole forest. This method was unbiased since the prediction for each sample was based on trees built from a subset of samples that did not include that sample. When the full forest was grown, the class predictions were compared to the true classes, generating the “OOB error rate” as a measure of prediction accuracy. Thus, the prediction accuracy was an unbiased estimate of how well one can predict sample class in a new data set. To determine which biochemicals made the largest contribution to the classification, a “variable importance” measure was computed. The “Mean Decrease Accuracy” (MDA) was used as this metric. The MDA was determined by randomly permuting a variable, running the observed values through the trees, and then reassessing the prediction accuracy.

#### Total serum and erythrocyte fatty acids

Serum and red blood cell membrane fatty acid profiles were performed by the Genetics Laboratories at the Kennedy Krieger Institute. Fatty acids were analyzed by capillary gas chromatography/mass spectrometry of pentaflourobenzyl bromide fatty acid esters using an AT-Silar-100 column (Grace, Columbia, Maryland 21044) as previously described [20]. For red blood cells only, the lipids were extracted with hexane:isopropanol before analysis. Standards and a year of quality control data were used to determine the average coefficient of variation percent (CV%) from RBC and plasma controls. Each run was required to pass clinical laboratory quality control before the data were released. CV% were typically under 10%.

#### Fatty acid content in fish

Fish fatty acid profiles were performed by Covance Laboratories (Madison, Wisconsin 53703). Each of the following fish types, fed to dolphins during the study, was mixed with water and homogenized for uniformity: capelin from Canada and Iceland (*Mallotus villosus*), Atlantic croaker (*Micropogonias undulatus*), herring (*Clupea harengus*), and striped mullet (*Mugil cephalus*). The lipid was extracted, saponified with 0.5N methanolic sodium hydroxide, and methylated with 14% BF3-methanol. The resulting methyl esters of the fatty acids were extracted with heptane. An internal standard was added prior to the lipid extraction. The methyl esters of the fatty acids were analyzed by gas chromatography using external standards for quantitation. Iron was measured by ICP Emission Spectrometry according to the Official Methods of Analysis of AOAC INTERNATIONAL, 18th Ed., Method 984.27 and 985.01, AOAC INTERNATIONAL, Gaithersburg, Maryland, USA, (2005). (Modified) (Covance, Madison, Wisconsin 53703). Dietary intake of nutrients, including fatty acids, were calculated for each dolphin, based on weighed daily breakout of fish (by fish and lot number) and most recent weight of each dolphin.

#### Statistics

Statistical analyses with the blood-based clinical and fatty acid data were conducted using SAS (SAS Inc., Cary, North Carolina). Dietary intake, hematology, clinical chemistries, fatty acids, and lipid profiles were compared in each group between baseline (Month 0) and subsequent treatment Months 1, 3 and 6 using Wilcoxon rank sum tests. Fatty acids that differed between the baseline and treatment months in the modified diet group and not the control group were included in stepwise logistic regression models to identify independent predictors of key clinical outcomes from the study (i.e. lower insulin and cholesterol and raised hematocrit and hemoglobin). Independent predictors were then assessed for linear associations using simple linear regression. Due to the prioritization of C15:0 from the metabolomic, clinical, and fatty acid analyses, simple linear regression was conducted using baseline (Month 0) data from the study population to assess potential relationships between C15:0 serum levels and age.

Baseline risk and protective factors for anemia were evaluated. Comparisons of age and sex by the presence or absence of anemia were assessed using a general linear model and two-sided Fisher’s exact test, respectively. Stepwise regression models were used to evaluate independent blood-based biomarker predictors of hemoglobin among all study dolphins at baseline, as well as study dolphins while on the modified diet. Biomarkers with a P value ≤ 0.05 in the stepwise regression model were subsequently evaluated for significant differences between dolphins with or without anemia using Wilcoxon two-sample test and two-sided P value. Across all studies, significance was defined as a P value ≤ 0.05.

## Results

There were no differences in age, sex, or body weight levels when comparing case and control groups at Month 0 (Table 1).

**Table 1:** Comparisons of demographics between feeding study case and control dolphins.

Demographic	Case dolphins on modified diet (n = 20)	Control dolphins remaining on baseline diet (n = 10)	*P* value
Mean age (years)	22 ± 14	26 ± 10	0.41
Sex (no. and % female)	9/20 (45%)	4/10 (40%)	0.79
Body weight (lbs)	389 ± 49	402 ± 45	0.33
Iron overload severity (ferritin level ng/ml)	5620 ± 5417	4574 ± 4121	0.28

### The modified diet lowered cholesterol and insulin in dolphins

At the beginning of the study, there were no differences in clinical chemistry values when comparing the modified diet and control groups, with the exception of lower albumin and higher erythrocyte sedimentation rates in the modified diet group (Table 2). As such, these variables were excluded as potential variables affected by the diet. By Month 1, dolphins on the modified diet had lower cholesterol and insulin compared to baseline diet controls. These changes in the modified diet group persisted through Month 6 and were not present in the control groups.

**Table 2:** Comparisons of clinical chemistry values in bottlenose dolphins (*Tursiops truncatus*) fed a modified versus baseline diet over 6 months.

Blood-based health indices	Month 0		Month 1		Month 3		Month 6	
	Modified Diet (n = 20)	Baseline Diet (n = 10)	Modified Diet (n = 20)	Baseline Diet (n = 10)	Modified Diet (n = 20)	Baseline Diet (n = 10)	Modified Diet (n = 20)	Baseline Diet (n = 10)
Ferritin (ng/ml)	5620±5417	4574±4121	3908±2794*	2181±1907	3888±2635	2289±1981	5495±7847	5615±5181
Insulin (μIU/ml)	9.8±6.1	9.4±4.8	7.5±4.0*	14.8±14.0	8.9±4.4*	16.3±13.6	6.8±4.6*	10.3±6.2
Glucose (mg/dl)	94±11	98±32	95±8	97±7	93±10	99±16	88±8	89±9
BUN (mg/dl)	46±5	45±6	49±5*	43±4	51±6*	43±6	53±8*	48±7
Creatinine (mg/dl)	1.3±0.2	1.3±0.2	1.2±0.2	1.3±0.2	1.2±0.3	1.3±0.2	1.1±0.2	1.3±0.2
Albumin (g/dl)	4.8±0.2*	5.0±0.2	4.7±0.3*	5.1±0.4	4.7±0.3*	5.2±0.3	4.6±0.2*	5.0±0.3
Alkaline phosphatase (IU/L)	381±165	323±183	360±157	329±170	324±163	322±149	285±136	311±150
AST (IU/L)	284±61	356±213	241±55	277±75	288±140	278±81	315±152	353±127
ALT (IU/L)	40±20	48±37	35±10	33±7	40±22	36±13	42±19	44±13
GGT (IU/L)	40±20	39±14	38±12	34±9	37±10	35±10	42±10	43±14
Total cholesterol (mg/dl)	183±35	187±23	160±26*	186±24	167±28*	199±33	160±31*	188±28
HDL cholesterol (mg/dl)	155±27	160±17	145±22*	162±22	149±20*	175±31	141±23*	165±27
VLDL cholesterol (mg/dl)	29±8	30±11	24±8*	32±10	28±20	28±10	24±8	28±9
Triglycerides (mg/dl)	144±40	147±56	122±42*	158±51	141±54	142±51	122±42	133±51
Iron (μg/dl)	190±74	187±62	185±74	174±55	208±82	195±65	235±63	201±75
Erythrocyte sedimentation rate (mm/hr)	10±11*	3±2	5±6	3±2	5±6	4±2	6±6	4±2
Uric acid (mg/dl)	0.67±0.28	0.68±0.19	0.69±0.33	0.59±0.30	0.74±0.35	0.70±0.23	0.81±0.30	0.78±0.25
Sodium (mEq/L)	157±2	158±2	159±2	158±3	158±2*	156±2	157±2	158±2
Potassium (mEq/L)	4.0±0.3	4.0±0.3	4.0±0.3	4.0±0.3	3.8±0.2	3.8±0.3	4.1±0.3	4.1±0.3
Chloride (mEq/L)	117±2	117±2	118±2	117±4	117±2*	115±2	117±3	116±1.4
CO2 (mEq/L)	26±2	28±2	27±3	27±1	29±1	28±2	27±2	27±2
Protein (g/dl)	6.7±0.6	6.8±0.4	6.7±0.5	6.7±0.5	6.6±0.5*	6.9±0.2	6.7±0.4*	7.0±0.4
Calcium (mg/dl)	9.0±0.2	8.9±0.3	9.0±0.3*	8.8±0.4	9.1±0.3	9.1±0.3	9.0±0.3	8.9±0.4
Inorganic phosphate (mg/dl)	4.7±0.6	4.7±0.4	5.0±0.3*	4.5±0.5	4.6±1.0	4.6±0.5	4.7±0.6	5.1±0.5
Lactate dehydrogenase (U/L)	635±82	617±90	618±71*	540±73	623±290*	527±81	613±78*	567±63
Creatine phosphokinase (U/L)	147±35	134±33	126±27	122±23	317±783*	119±34	149±37	138±27
Magnesium (mg/dl)	2.1±0.2	2.0±0.1	2.0±0.2	2.0±0.1	1.9±0.2	2.0±0.3	1.9±0.1	1.9±0.2
Anion gap (mEq/L)	14±3	14±3	14±2	14±2	12±2	14±2	13±2*	15±2

*Wilcoxon rank sum P-value ≤ 0.05 compared to Month 0.

### The modified diet alleviated anemia in dolphins

**Fig 1:**
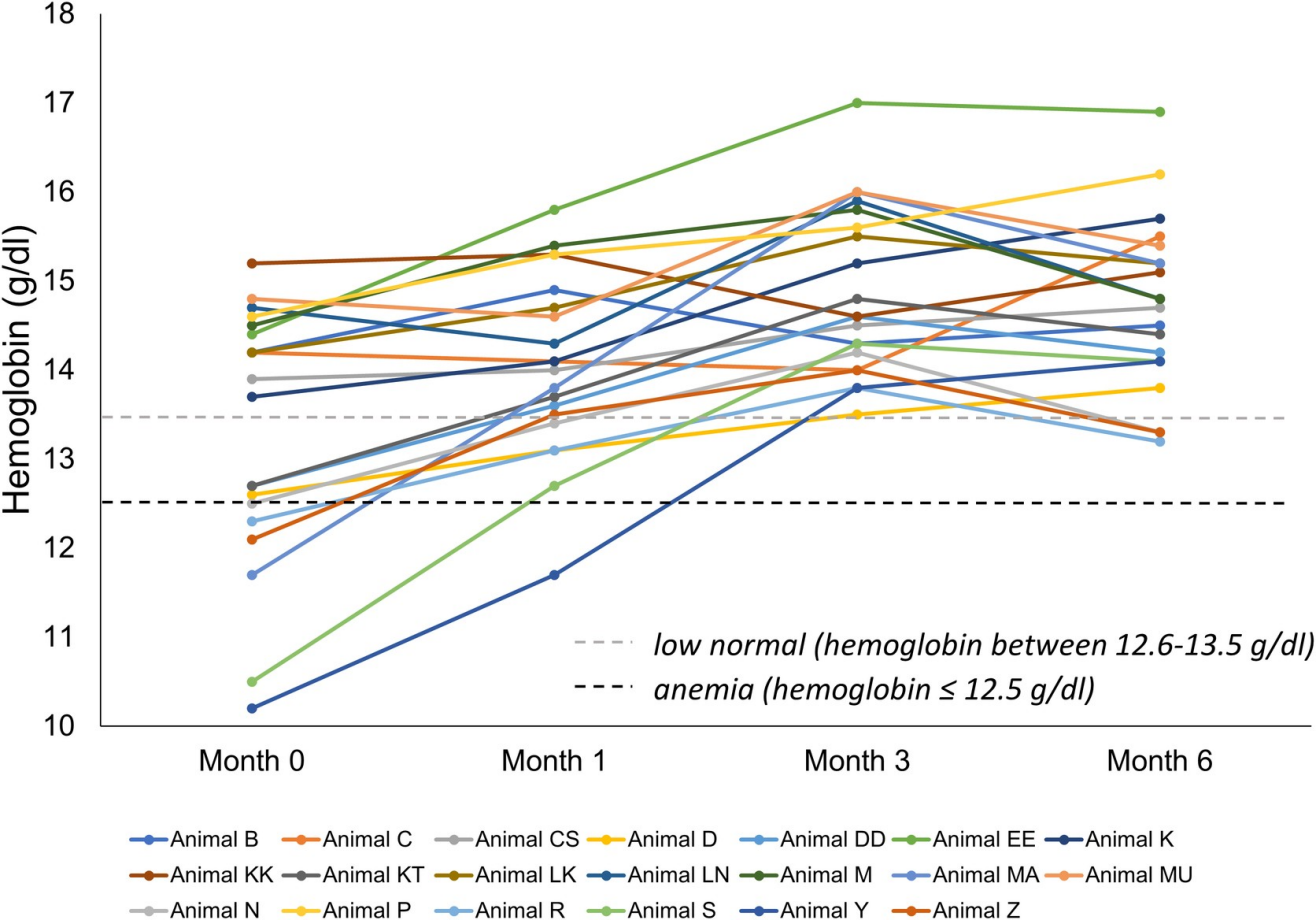
Alleviated anemia (anemia defined as hemoglobin ≤ 12.1 g/dl) and raised low-normal hemoglobin (low-normal hemoglobin defined as 12.2–13.2 g/dl) in bottlenose dolphins (*Tursiops truncatus*) within three months on a modified fish diet.

At the beginning of the study, there were no differences in hematology values when comparing the modified diet and control groups, except for higher WBC counts (Table 3). As such, WBC count was excluded as a potential variable affected by the diet. By Month 1, dolphins on the modified diet had higher hematocrit, platelets, and packed cell volume compared to the controls. By Month 3, dolphins on the modified diet also had higher hemoglobin and red blood cells, as well as lower red blood cell distribution width, compared to the controls. These changes in the modified diet group persisted through Month 6 and were not present in the control groups. All dolphins with anemia (defined as hemoglobin ≤ 12.1 g/dl, n = 6) or low-normal hemoglobin (12.2–13.2 g/dl, n = 3) at Month 0 that were placed on the modified diet had resolved, normal hemoglobin (> 13.2 g/dl) by Month 3 (Fig 1).

**Table 3:** Comparisons of red and white blood cell index values in bottlenose dolphins (*Tursiops truncatus*) fed a modified versus baseline diet over 6 months.

Variable	Month 0		Month 1		Month 3		Month 6	
	Modified Diet	Baseline Diet	Modified Diet	Baseline Diet	Modified Diet	Baseline Diet	Modified Diet	Baseline Diet
Hemoglobin (g/dl)	13±1	13±1	14±1	13±1	15±1*	14±1	15±1*	13±1
Hematocrit (%)	44±5	44±3	46±3*	44±3	47±2*	45±2	47±2*	44±2
Red blood cell distribution width (%)	15±2	15±2	14±1	15±2	13±1*	15±2	13±1*	16±3
Platelets (x10^9^/L)	90±29	79±30	103±26*	80±30	101±24	86±37	95±27	80±38
White blood cells (x10^9^/L)	8.0±2.1	6.8±0.9	8.0±1.8	6.4±1.5	8.2±2.6	7.0±2.2	8.0±2.3	7.2±2.1
Red blood cells (x10^6^/L)	3.0±0.3	3.0±0.2	3.2±0.2	3.0±0.2	3.4±0.2*	3.2±0.2	3.3±0.3*	3.1±0.2
Mean corpuscular volume (fl)	145±5	145±5	144±5	143±5	142±5	141±5	141±5	142±6
Mean corpuscular hemoglobin (pg)	44±1	44±2	44±2	44±2	44±2*	43±2	44±2	43±2
MCHC (g/dl)	31±1	30±1	31±0.8	30±1	31±1*	30±1	31±1*	30±1
Nucleated RBCs (x10^3^/μL)	1.8±2.9	1.4±2.5	0.5±0.9*	1.2±1.5	0.2±0.6*	1.1±1.0	0.2±0.5	0.7±1.3
Lymphocytes (x10^3^/μL)	0.77±0.48	0.61±0.50	0.71±0.41*	0.51±0.47	0.64±0.27	0.51±0.34	0.73±0.36	0.68±0.29
Neutrophils (x10^3^/μL)	5.8±1.8	5.0±1.2	5.8±1.7*	4.6±1.3	6.0±2.4	5.0±1.6	5.8±1.7	5.3±1.9
Monocytes (x10^3^/μL)	0.39±0.28	0.23±0.11	0.40±0.22	0.28±0.16	0.42±0.23	0.35±0.28	0.39±0.31	0.28±0.18
Eosinophils (x10^3^/μL)	1.1 ± 0.6	1.0±0.4	1.1±0.5	1.0±0.5	1.1±0.5	1.1±0.7	1.1±0.4	0.9±0.3
Packed cell volume (%)	38±4	38±3	41±2*	39±2	42±2*	40±3	42±2*	39±2

*Wilcoxon rank sum P-value ≤ 0.05 compared to Month 0.

### The modified diet shifted the serum metabolome

**Fig 2:**
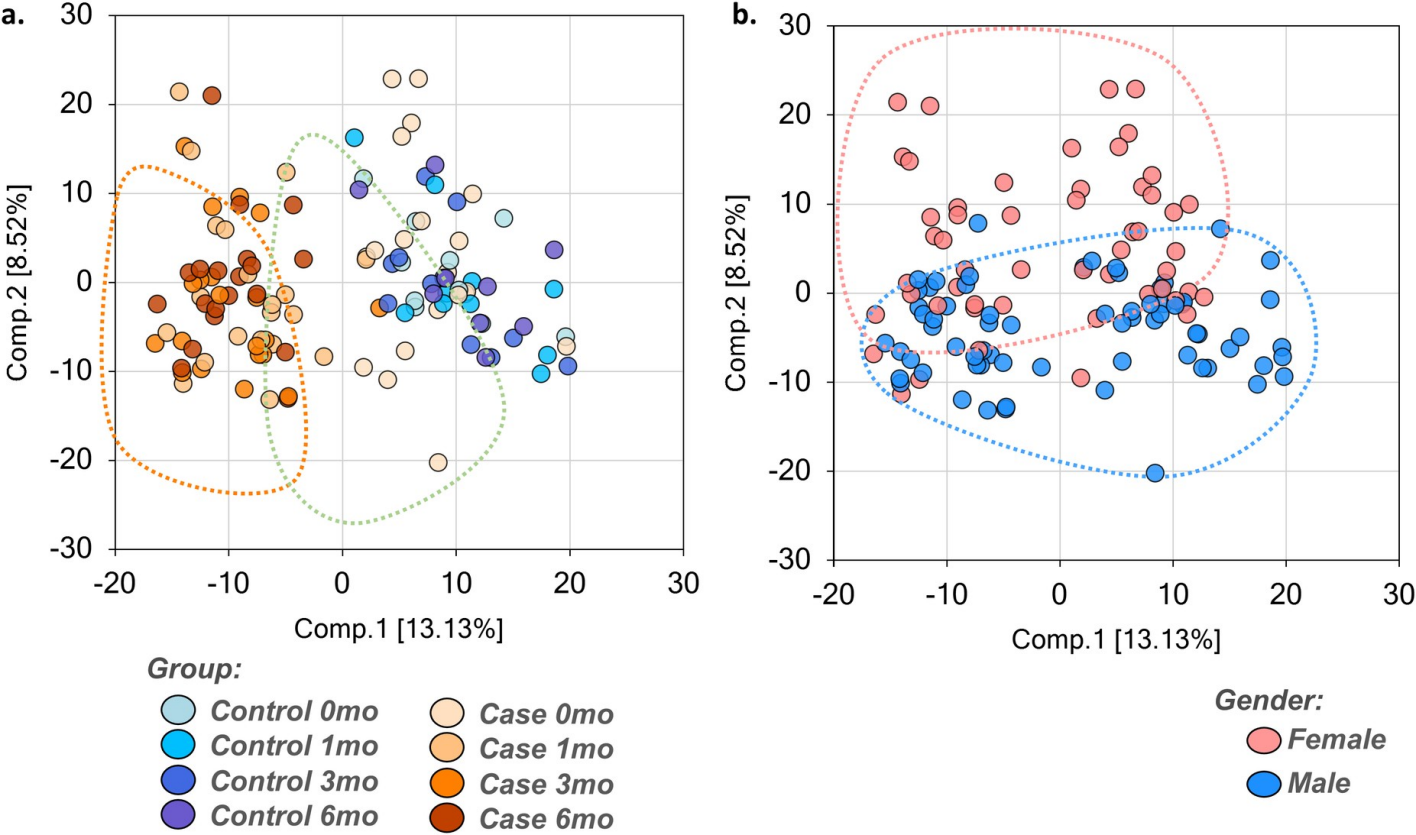
Principal component analyses involving 819 biochemicals detected in the dolphin serum metabolome, including demonstrated (a) shift of the metabolome among dolphins on the modified wild-type fish diet (‘Case’) compared to dolphins on the baseline fish diet (‘Control’), and (b) some separation of metabolites between females and males.

**Fig 3:**
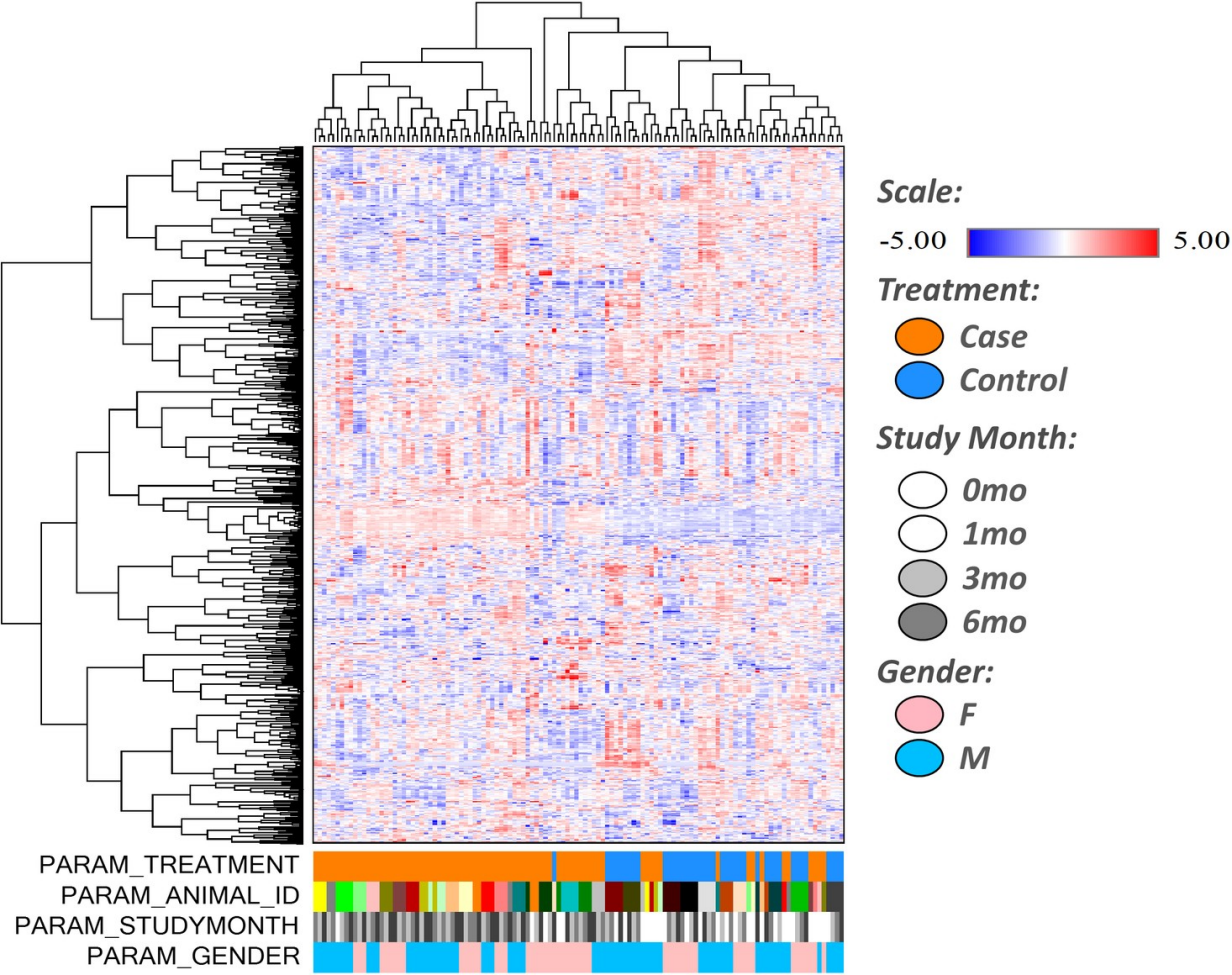
Hierarchical clustering analysis involving 819 biochemicals detected in the dolphin serum metabolome, including large-scale effects of the metabolome among dolphins on the modified wild-type fish diet (‘Case’) compared to dolphins on the baseline fish diet (‘Control’) (solid orange bar).

A total of 819 small molecule biochemicals were measured in dolphin serum, of which 664 compounds had known identity and 155 had unknown structural identity. Detected serum biochemicals included amino acids, peptides, carbohydrates, lipids, nucleotides, cofactors and vitamins, and xenobiotics across over 60 pathways. Of detected biochemicals in the dolphin serum, 241 (29.4%) were characterized as changed (p ≤ 0.05) due to the main effect of the modified diet. Principal component analysis demonstrated a clear shift in the dolphin metabolome by Month 1 on the modified diet that was sustained throughout the six-month study (Fig 2A). There were also differences in the metabolome when comparing females and males (Fig 2B). Hierarchical clustering demonstrated similar large-scale effects of the modified diet on the dolphin metabolome (Fig 3).

### Serum OCFAs were the leading biochemical predictors of the modified diet

At baseline (Month 0), differences in metabolites detected between the Modified Diet and Control groups were 50% accurate, which is equal to random chance (Table 4). The top 30 prioritized biochemicals that changed as a result of the modified fish diet at Month 1 and Month 3, however, were 100% predictive of which dolphins were on the modified diet. Of the 30 prioritized biochemicals at Month 1, 25 (83%) were lipids, and of the lipids, 10/25 (40%) contained odd-chain saturated fatty acids (OCFAs), including C15:0, C17:0 and C19:0. C15:0, pentadecanoic acid, was the highest-prioritized OCFA during Months 1 and 3.

**Table 4:** Categories of the top 30 prioritized serum biochemicals, based on random forest regression, that changed in the bottlenose dolphin (*Tursiops truncatus*) metabolome due to the modified fish diet.

Biochemical or Biochemical Group	Placement or numbers in the biochemical importance plot (top 30 compounds) related to the modified diet vs. control groups		
	Month 0	Month 1	Month 3
**Prioritization placement of OCFAs**			
C15:0	none	9th	5th
C17:0	none	13th	11th
C19:0	none	24th	27th
**Number of biochemicals in top 30 list**			
Amino acids	7	2	2
Carbohydrate	1	0	0
Cofactors and vitamins	0	0	0
Complex lipids	0	0	0
Energy	0	0	0
Lipids	12	25	23
- OCFAs and OCFA-containing lipids	0	10	9
Nucleotides	4	0	0
Peptides	0	0	0
Xenobiotics	1	0	0
Unknown biochemicals	5	3	5
**Predictive accuracy of importance plot to group**	50%*	100%	100%

*50% predictive accuracy is equal to random chance

### The modified diet increased daily OCFA intake

The modified diet did not change total daily kilocalories, pounds of fish, moisture, or carbohydrates ingested compared to the baseline (Table 5). The modified diet did increase the daily intake of protein, ash, OCFAs (C15:0 and C17:0), omega-3 fatty acid C18:3 (α-linolenic acid), overall omega-6 fatty acids (including C20:4n6, arachidonic acid),

**Table 5:** Comparisons of average daily nutrient and fatty acid intake in bottlenose dolphins (*Tursiops truncatus*) fed a modified versus baseline diet over 6 months.

Daily Nutrient Intake	Case Dolphins		Control Dolphins	
	Baseline diet (pre-study)	Modified diet	Baseline diet (pre-study)	Baseline diet (during study)
Total kilocalories	8,923 ± 2,411	9,139 ± 2,171	8,442 ± 1,527	7,702 ± 2,897
Total pounds	16 ± 4	16 ± 4	15 ± 3	15 ± 3
Total moisture (g)	5,488 ± 1,548	5,394 ± 1,301	5,234 ± 972	5,261 ± 904
Total protein (g)	1,014 ± 281	1,188 ± 286*	965 ± 177	981 ± 180
Total fat (g)	494 ± 133	460 ± 111*	468 ± 87	457 ± 89
Total carbohydrates (g)	25 ± 11	21 ± 5	20 ± 6	20 ± 6
Total ash (g)	145 ± 40	297±71*	138 ± 25	143 ± 27
Fatty acids (g)				
C12:0	0.1 ± 0.04	0.1 ± 0.04	0.1 ± 0.02	0.1 ± 0.08
C14:0	23 ± 7	15 ± 4*	22 ± 4	20 ± 4
C14:1	0.1 ± 0.04	0.1 ± 0.04	0.1 ± 0.02	0.1 ± 0.08
C15:0	1.3 ± 0.4	4.5 ± 1.1*	1.2 ± 0.2	1.2 ± 0.3
C16:0	58 ± 17	48 ± 14*	55 ± 9	53 ± 14
C16:1	38 ± 11	24 ± 6*	36 ± 6	34 ± 6
C17:0	0.3 ± 0.1	1.1 ± 0.3*	0.3 ± 0.1	0.4 ± 0.2
C18:0	6.4 ± 1.9	7.6 ± 2.0	6.0 ± 0.9	6.1 ± 1.8
C18:2	3.6 ± 1.2	2.7 ± 0.8*	3.4 ± 0.7	3.2 ± 0.7
C18:3 n3	0.2 ± 0.1	0.6 ± 0.2*	0.2 ± 0.04	0.2 ± 0.2
C18:3 n6	1.5 ± 0.5	1.3 ± 0.4*	1.4 ± 0.2	1.5 ± 0.5
C18:4	5.1 ± 1.5	3.4 ± 1.0*	4.8 ± 0.8	4.6 ± 0.9
C20:0	0.8 ± 0.2	0.4 ± 0.1*	0.7 ± 0.1	0.7 ± 0.2
C20:1	49 ± 15	19 ± 5*	47 ± 9	43 ± 11
C20:2	0.2 ± 0.1	0.1 ± 0.1*	0.2 ± 0.1	0.2 ± 0.1
C20:3 n3	0.1 ± 0.1	0.1 ± 0.04	0.2 ± 0.1	0.1 ± 0.1
C20:4 n6	1.6 ± 0.5	3.6 ± 0.9*	1.5 ± 0.2	1.5 ± 0.6
C20:5	40 ± 12	28 ± 8*	38 ± 6	36 ± 7
C22:0	0.1 ± 0.05	0.1 ± 0.04	0.1 ± 0.02	0.1 ± 0.1
C22:1	8.0 ± 2.5	3.0 ± 0.7*	7.6 ± 1.4	7.1 ± 2.0
C22:5	3.8 ± 1.1	4.8 ± 1.2*	3.6 ± 0.6	3.6 ± 0.7
C22:6	28 ± 8	21 ± 6*	27 ± 4	27 ± 5
C24:0	0	0.3 ± 0.1*	0	0
C18:1 n9	38 ± 12	29 ± 10*	25 ± 6	33 ± 15
Total monounsaturated fats	144 ± 43	83 ± 23*	137 ± 23	128 ± 24
Total omega 3	81 ± 23	60 ± 17*	77 ± 12	75 ± 15
Total omega 6	6 ± 2	7 ± 2*	5 ± 1	5 ± 2
Total omega 9	97 ± 29	52 ± 15*	93 ± 16	86 ± 16
Total polyunsaturated fats	84 ± 24	64 ± 18*	79 ± 13	77 ± 16
Total saturated fats	86 ± 25	73 ± 20*	81 ± 13	78 ± 19
Total 18:1 cis	53 ± 16	39 ± 13*	50 ± 8	47 ± 18
Total 18:1 trans	3.3 ± 1.0	1.5 ± 0.4*	3 ± 0.6	2.9 ± 0.6
Total 18:2 trans	0.8 ± 0.2	1.0 ± 0.3*	0.7 ± 0.1	0.7 ± 0.2
Total 18:3 trans	6.6 ± 2.5	3.1 ± 1.0*	6.4 ± 1.7	5.4 ± 1.1
Total cis unsaturated fats	227 ± 66	147 ± 41*	216 ± 36	205 ± 39
Total fatty acids	338 ± 99	236 ± 66*	320 ± 53	305 ± 61
Total trans fats	10 ± 3	5 ± 2*	10 ± 2	9 ± 2

*Wilcoxon rank sum P-value ≤ 0.05 compared to Month 0.

C22:5 (docosapentaenoic acid), and the even-chained saturated fatty acid C24:0 (lignoceric acid). The modified diet decreased daily intake of total fat and several even-chained saturated fatty acids (C14:0, C16:0, and C20:0). The modified diet also decreased daily intake of total omega-3 fatty acids (including C20:5n3 and C22:6n3), total ω-9 fatty acids, and total fatty acids (including total monounsaturated fatty acid, polyunsaturated fatty acids, saturated fatty acids, and trans fats).

### The modified diet increased serum OCFAs

Within one month on the modified diet, changes in dietary fatty acid intake were reflected in dolphin serum fatty acid profiles (Table 6). The modified diet lowered total serum C14:0, C16:1n7, C16:1n9, C16:1T, C18:1n7, C18:1T, C20:1n9, C20:2n6, C20:5n3, C22:1n9, C22:6n3, C26:1, pristanic acid to phytanic acid ratio, pristanic acid, trans fats, total omega-3 fatty acids, and total omega-7 and omega-5 fatty acids. The modified diet increased C15:0, C17:0, C17:1, C18:2T, C18:3n6, C20:3n6, C20:3n9, C20:4n6, C22:4n6, C22:5n6, C25:0, C26:0, and arachidonic acid to docosahexaenoic acid ratio. These changes were not present in the control population.

**Table 6:** Comparisons of serum total fatty acid values in bottlenose dolphins (*Tursiops truncatus*) fed a modified versus baseline diet.

Serum Fatty Acids (μg/ml)	Modified Diet				Baseline Diet			
	Month 0	Month 1	Month 3	Month 6	Month 0	Month 1	Month 3	Month 6
C10:0	0.14±0.04	0.14±0.05	0.17±0.07	0.14±0.02	0.13±0.02	0.13±0.02	0.15±0.02*	0.14±0.02*
C10:1	0.01±0.00	0.01±0.01	0.01±.000	0.01±0.01*	0.01±0.00	0.01±0.01*	0.01±0.00	0.01±0.01
C12:0	0.91±0.38	0.82±0.27	1.03±0.36	0.69±0.24*	0.83±0.17	0.86±0.21	1.11±0.21*	0.73±0.17
C12:1	0.01±0.00	0.01±0.00	0.02±0.01*	0.01±0.00	0.01±0.00	0.01±0.00	0.01±0.01	0.01±0.00
C13:0	0.34±0.08	0.42±0.08*	0.53±0.17*	0.40±0.13	0.35±0.08	0.37±0.11	0.35±0.08	0.34±0.09
C14:0	73±15	55±14*	62±17*	50±13*	76±17	76±16	76±12	74±17
C14:1	2.5±0.8	2.4±1.5	3.2±2.3	2.0±1.2*	2.5±0.7	2.8±0.9	3.5±1.1*	2.1±0.6
C14:2	0.03±0.01	0.03±0.02	0.04±0.02	0.03±0.01	0.03±0.01	0.03±0.01	0.03±0.01	0.03±0.01
C15:0	7±1	28±9*	34±11*	33±10*	8±1	8±2	8±1	8±2
C16:0	351±41	327±52*	368±70	328±50*	362±63	371±63	395±68	369±56
C16:1n7	219±26	175±31*	194±55*	185±34*	239±50	228±46	238±36	229±46
C16:1n9	5.7±1.0	4.6±1.5*	5.4±1.9*	5.0±1.2*	6.1±1.8	6.1±1.8	6.8±1.5	6.2±1.7
C16:1T	24±4	13±3*	14±3*	15±3*	24±4	24±4	25±4	28±5
C16:2	1.2±0.4	1.2±0.8	0.4±0.3*	1.1±0.6	1.3±0.3	1.2±0.6	0.5±0.3*	0.8±0.7
C17:0	8±1	23±5*	28±8*	25±9*	8±2	9±2	9±2	8±2
C17:1	5±1	28±7*	34±9*	32±8*	5±1	7±2	6±1	6±1
C18:0	180±27	166±22*	194±45	163±23*	196±40	206±44	220±44	193±41
C18:1n5	0.06±0.04	0.05±0.04	0.07±0.07	0.04±0.04*	0.07±0.04	0.06±0.04	0.05±0.03	0.08±0.06
C18:1n7	78±12	66±17*	73±19*	69±12*	84±21	80±21	83±16	82±17
C18:1n9	444±66	390±78*	429±81	417±51*	465±91	439±86	474±71	454±17
C18:1T	52±9	26±7*	27±11*	28±7*	58±16	55±11	54±9	56±11
Conjugated C18:2n6 (rumenic acid)	30±3	23±7*	29±7*	24±3*	33±8	34±9	38±5*	34±7*
C18:2n6	0.36±0.07	0.31±0.09*	0.35±0.09	0.36±0.07*	0.36±0.10	0.34±0.09	0.36±0.06*	0.36±0.07
C18:2T	1.9±0.2	3.2±1.1*	3.7±0.6*	3.4±0.9*	1.9±0.5	2.2±0.6	2.0±0.3	2.0±0.4
C18:3n3	6.4±1.3	6.2±2.0	7.3±2.4	6.0±1.9*	6.7±2.2	7.5±2.4	7.3±1.5	6.3±1.6
C18:3n6	2.8±0.4	4.3±0.9*	4.9±1.0*	4.4±1.1*	3.0±0.5	3.1±0.7	3.0±0.5	3.2±0.8
C20:0	25±36	25±4	26±5	22±4	24±3	24±2	27±5	22±4
C20:1n9	118±36	65±26*	69±25*	67±28*	124±38	125±38	120±33	146±54
C20:2n6	1.9±0.4	1.4±0.4*	1.6±0.4*	1.4±0.3*	2.0±0.6	2.1±0.5	2.0±0.3	2.0±0.6
C20:3n6	3.1±0.5	5.6±1.0*	6.3±1.4*	5.9±0.8*	3.3±0.8	3.4±0.9	3.5±0.7	3.4±0.8
C20:3n7	0.24±0.03	0.33±0.06*	0.40±0.05*	0.38±0.07*	0.24±0.04	0.28±0.05	0.31±0.04*	0.30±0.06*
C20:3n9	0.15±0.03	0.26±0.05*	0.30±0.07*	0.33±0.07*	0.17±0.03	0.14±0.03	0.13±0.02*	0.13±0.02*
C20:4n6	91±15	198±32*	214±31*	192±33*	93±13	96±14	99±11	90±17
C20:5n3	480±77	302±57*	343±63*	320±69*	508±86	486±94	499±72	492±114
C21:0	2.2±0.5	3.3±0.6*	3.9±0.8*	3.4±0.8*	2.1±0.4	2.0±0.3	2.5±0.6*	2.0±0.5
C22:0	4.7±1.0	6.5±1.0*	7.8±1.3*	6.5±1.2*	4.5±0.5	4.5±0.4	5.4±0.9*	4.5±0.7
C22:1n9	16±5	8±4*	10±4*	10±4*	16±5	18±6	18±7	19±6
C22:2n6	0.32±0.08	0.27±0.05*	0.27±0.07*	0.29±0.10	0.33±0.09	0.35±0.08	0.38±0.08	0.40±0.10
C22:4n6	1.4±0.3	5.1±1.1*	6.1±1.3*	6.9±1.3*	1.4±0.3	1.3±0.2	1.5±0.3	1.6±0.3
C22:5n3	63±14	65±15	71±20	74±16*	64±21	61±17	57±18	68±20
C22:5n6	2.4±0.5	4.9±0.9*	5.6±1.0*	5.4±1.0*	2.5±0.9	2.6±0.8	2.8±0.6	2.7±1.0
C22:6n3	196±30	157±29*	171±31*	166±27*	216±49	215±43	214±27	212±50
C23:0	1.3±0.3	3.4±0.7*	4.4±0.8*	3.6±0.7*	1.3±0.2	1.3±0.2	1.6±0.3*	1.4±0.2
C24:0	2.5±0.5	5.8±1.0*	7.4±1.5*	6.2±1.3*	2.4±0.3	2.2±0.3	2.9±0.5*	2.6±0.4
C24:1n9	55±9	35±6*	40±6*	36±5*	52±6	48±5*	59±10	54±9
C24:2	0.31±0.06	0.43±0.07*	0.44±0.07*	0.41±0.06*	0.30±0.07	0.32±0.05	0.35±0.06*	0.32±0.07*
C25:0	0.06±0.01	0.17±0.02*	0.20±0.04*	0.20±0.04*	0.06±0.01	0.06±0.01	0.07±0.01	0.07±0.01
C25:1	0.31±0.05	0.31±0.04	0.33±0.07	0.34±0.05*	0.29±0.06	0.28±0.04	0.32±0.06	0.30±0.08
C26:0	0.09±0.02	0.14±0.02*	0.15±0.04*	0.14±0.03*	0.09±0.01	0.10±0.04	0.10±0.02	0.09±0.01
C26:1	0.46±0.11	0.37±0.11*	0.39±0.11*	0.35±0.08*	0.43±0.10	0.47±0.15	0.49±0.15	0.50±0.10
C26:2	0.02±0.01	0.02±0.01*	0.03±0.01*	0.02±0.00*	0.02±0.00	0.03±0.01*	0.03±0.01*	0.02±0.01
C28:0	0.01±0.00	0.01±0.01	0.01±0.00	0.03±0.01*	0.02±0.00	0.01±0.00	0.01±0.00*	0.02±0.01
C29:0	0.01±0.00	0.01±0.01	0.01±0.00	0.02±0.01*	0.01±0.00	0.01±0.00	0.01±0.00	0.01±0.01
C30:0	0.01±0.00	0.01±0.00*	0.01±0.00	0.02±0.01*	0.01±0.00	0.01±0.00*	0.01±0.00	0.01±0.01
Phytanic acid	18±7	16±5	20±6	17±5	14±5	16±5	18±4*	15±4*
Pristanic to phytanic acid ratio	0.23±0.04	0.16±0.03*	0.17±0.03*	0.18±0.04*	0.26±0.08	0.26±0.08	0.27±0.09	0.31±0.06
Pristanic acid	3.9±0.9	2.5±0.9*	3.3±1.0*	2.9±0.8*	3.5±0.8	3.9±1.3	4.5±1.2*	4.5±0.9
Total fatty acids	2582±316	2251±352*	2529±452*	2341±314*	2716±492	2674±473	2794±352	2706±471
Total saturated fats	656±77	640±85	739±139*	644±88	686±118	705±118	750±118	685±110
Total trans fats	77±12	42±10*	45±14*	47±10*	84±20	81±15	81±12	86±16
Total omega-3	745±108	531±94*	593±109*	565±104*	794±150	769±142	779±106	778±177
Total omega-7,5	302±36	243±48*	271±75*	256±45*	325±72	311±67	324±51	313±62
Total omega-9	645±96	532±105*	589±110	568±68*	667±129	644±124	686±88	687±128
Arachidonic to DHA ratio	0.5±0.1	1.3±0.2*	1.3±0.1*	1.2±0.2*	0.4±0.1	0.5±0.1	0.5±0.1	0.4±0.1

*Wilcoxon rank sum P-value ≤ 0.05 compared to Month 0.

### The modified diet increased erythrocyte membrane OCFAs

Changes in dietary fatty acid intake were reflected in dolphin erythrocyte fatty acid profiles (Table 7). Dolphins on the modified diet had lower erythrocyte fatty acid levels of C14:0, C16:1n9, C16:1T, C18:1T, C20:1n9, C20:2n6, C20:5n3, C22:1n9, pristanic acid, C18:1 dimethylacetal, total trans fatty acids, total omega-3 fatty acids, and C18:0 DMA to C18:0 ratio. Dolphins on the modified diet had higher erythrocyte fatty acid levels of C10:0, C15:0, C17:0, C17:1, C18:1n9, C18:2T, C18:3n6, C20:0, C20:3n6, C20:3n9, C20:4n6, C22:0, C22:4n6, C22:5n3, C22:5n6, C23:0, C24:0, C24:2, C25:0, C26:0, C26:1, C26:2, C28:0, total fatty acids, total saturated fatty acids, and arachidonic acid to docosahexaenoic acid ratio. These changes were not present in the control population.

**Table 7:** Comparisons of baseline and final erythrocyte total fatty acid values in bottlenose dolphins (*Tursiops truncatus*) fed a modified versus baseline diet.

RBC Fatty Acids (μg/ml)	Modified Diet		Baseline Diet	
	Month 0	Month 6	Month 0	Month 6
C10:0	0.08±0.01	0.10±0.03*	0.08±0.02	0.09±0.02
C10:1	0.01±0.00	0.01±0.01	0.01±0.00	0.01±0.01
C12:0	0.33±0.17	0.31±0.16*	0.30±0.06	0.26±0.08*
C12:1	0.01±0.01	0.01±0.00	0.01±0.01	0.01±0.01
C14:0	6.1±1.0	4.5±0.5*	6.5±1.2	6.8±1.1
C14:1	0.09±0.02	0.09±0.03	0.10±0.04	0.12±0.03
C14:2	0.01±0.02	0.00±0.01	0.01±0.01	0.00±0.01
C15:0	1.5±0.3	5.8±0.8*	1.6±0.2	1.6±0.2
C16:0	132±18	128±12	149±25	155±19
C16:1n7	15±2	14±2	16±3	20±4*
C16:1n9	0.37±0.07	0.33±0.05*	0.40±0.11	0.47±0.11
C16:1T	1.9±0.3	1.2±0.1*	2.0±0.5	2.4±0.5*
C16:2	0.04±0.02	0.07±0.01*	0.04±0.11	0.07±0.01*
C17:0	4±1	17±2*	5±1	5±1
C17:1	0.5±0.1	3.6±0.5*	0.5±0.1	0.5±0.1
C18:0	124±16	165±16*	145±17	163±18*
C18:1n5	0.02±0.01	0.03±0.02*	0.02±0.01	0.03±0.01*
C18:1n7	20±3	22±3*	21±4	26±6*
C18:1n9	81±13	71±11*	87±17	84±23
C18:1T	6±2	4±1*	8±2	9±2*
Conjugated C18:2n6 (rumenic acid)	4.4±0.6	3.6±0.5*	4.8±0.7	5.3±1.4*
C18:2n6	0.10±0.03	0.10±0.02*	0.11±0.03	0.10±0.03*
C18:2T	0.19±0.04	0.35±0.03*	0.20±0.05	0.24±0.06
C18:3n3	0.29±0.04	0.28±0.04	0.33±0.09	0.34±0.011
C18:3n6	0.14±0.03	0.22±0.02*	0.16±0.03	0.17±0.04
C20:0	14±3	20±2*	15±3	16±3
C20:1n9	50±7	30±4*	60±12	70±22
C20:2n6	0.77±0.012	0.61±0.07*	0.86±0.12	0.99±0.42
C20:3n6	1.2±0.2	2.1±0.3*	1.3±0.2	1.7±0.9
C20:3n7	0.24±0.05	0.26±0.05	0.26±0.06	0.31±0.10
C20:3n9	0.07±0.02	0.18±0.04*	0.07±0.02	0.07±0.03
C20:4n6	109±21	190±24*	114±19	123±61
C20:5n3	128±16	84±13*	150±28	177±66
C22:0	8±1	15±2*	9±1	10±1
C22:1n9	12±2	11±1*	15±3	19±5*
C22:2n6	0.13±0.02	0.14±0.01	0.14±0.02	0.15±0.06
C22:4n6	0.6±0.2	2.0±0.3*	0.5±0.1	0.5±0.3
C22:5n3	8±2	12±2*	8±2	10±4
C22:5n6	0.6±0.2	1.5±0.2*	0.6±0.1	0.8±0.5
C22:6n3	30±5	30±5	33±5	38±19
C23:0	2±1	7±1*	3±1	2±1
C24:0	6±2	18±2*	6±1	6±1
C24:1n9	109±14	115±12	119±17	142±53*
C24:2	0.5±0.1	0.9±0.1*	0.5±0.1	0.6±0.3
C25:0	0.2±0.1	1.1±0.2*	0.2±0.1	0.2±0.1
C26:0	0.13±0.03	0.38±0.06*	0.12±0.02	0.12±0.04
C26:1	4.4±1.0	8.4±2.1*	4.4±1.3	4.3±1.6
C26:2	0.03±0.01	0.07±0.01*	0.03±0.01	0.04±0.02
C28:0	0.01±0.00	0.02±0.01*	0.01±0.00	0.01±0.00
C30:0	0.01±0.01	0.01±0.00	0.01±0.01	0.01±0.01*
Phytanic acid	0.81±0.28	0.77±0.21	0.71±0.31	0.83±0.20
Pristanic acid	0.16±0.03	0.10±0.02*	0.16±0.04	0.20±0.03*
Total C18:1 DMA	1.8±0.4	1.2±0.2*	1.7±0.3	1.9±0.3
Total branched	1.0±0.3	0.9±0.2	0.9±0.3	1.0±0.2
Total DMA	6.3±1.1	5.8±0.8	6.2±1.1	6.8±0.9
Total fatty acids	891±105	997±84*	996±135	1110±292
Total saturated fats	300±38	382±30*	340±46	365±40
Total trans fats	8±2	6±1*	10±2	12±3
Total omega-3	166±21	126±18*	192±34	225±88
Total omega-7,5	34±5	36±4	38±7	46±10*
Total omega-9	258±31	240±25*	286±45	321±100
16DMA	2.8±0.5	2.9±0.4	2.8±0.5	3.1±0.4
16DMA:16	0.02±0.01	0.02±0.00	0.02±0.00	0.02±0.00
18DMA	1.7±0.4	1.8±0.3	1.7±0.3	1.9±0.3
18DMA:18	0.01±0.00	0.01±0.00*	0.01±0.00	0.01±0.00
Arachidonic to DHA ratio	3.7±0.6	6.4±0.9*	3.5±0.9	3.3±0.5

*Wilcoxon rank sum P-value ≤ 0.05 compared to Month 0.

### Changes in circulating fatty acids were associated with lowered cholesterol and insulin

**Fig 4:**
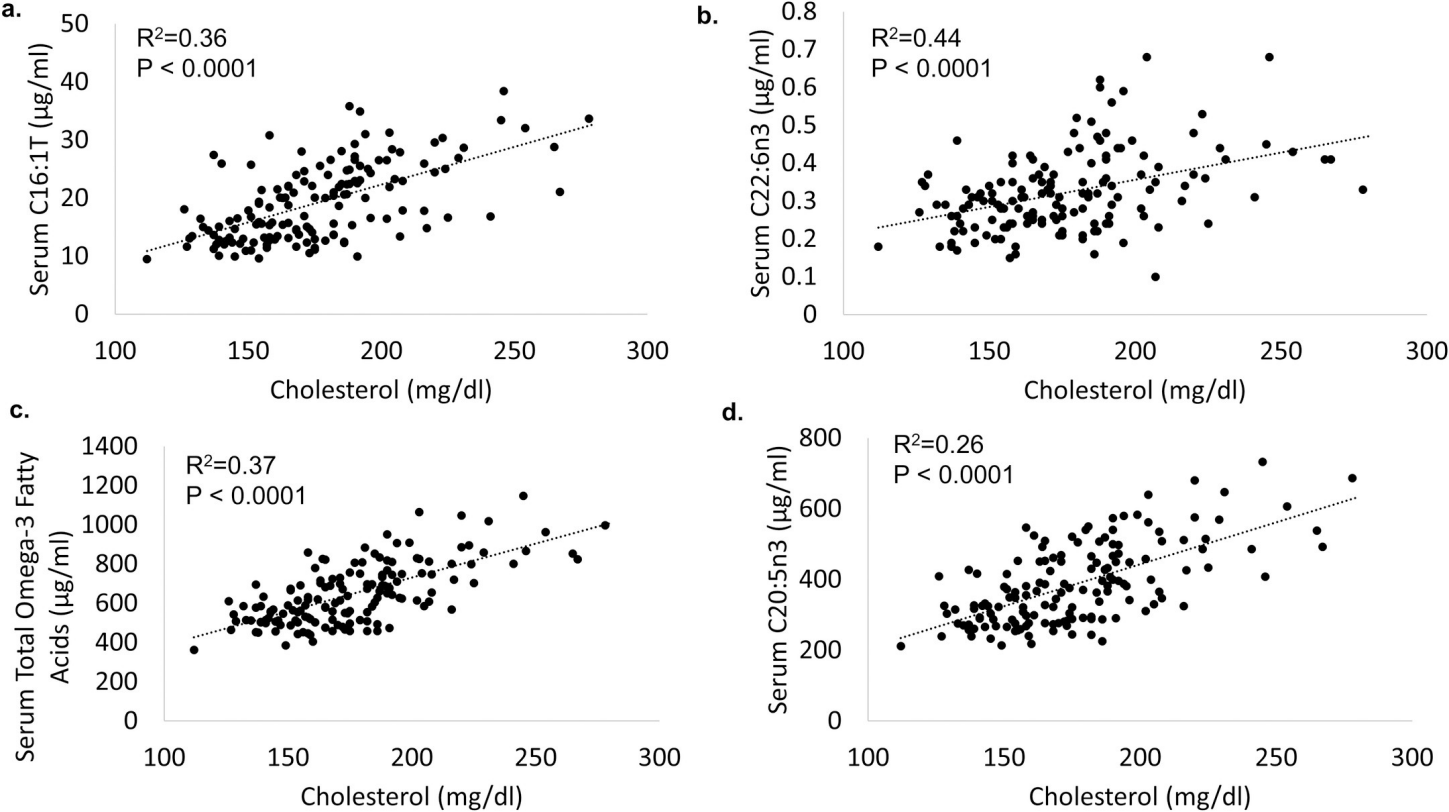
Independent and linear serum fatty acid predictors of cholesterol in bottlenose dolphins, including (a) C16:1T, (b) C22:6n3 (DHA), (c) total omega-3 fatty acids, and (d) C20:5n3 (EPA).

Lowered serum levels of C16:1n7, C16:1T, C18:1n7, C20:2n6, C20:5n3, C22:6n3, pristanic acid to phytanic acid ratio, pristanic acid, and total omega-3 fatty acids independently predicted lower cholesterol. Raised serum levels of C20:3n6, C25:0, and arachidonic acid to DHA ratios also predicted lower cholesterol. Of these, lowered serum levels of C16:1n7, C16:1T, C18:1n7, C20:2n6, C20:5n3, C22:6n3, pristanic acid, and total omega-3 fatty acids were independent, linear predictors of lower cholesterol (Table 8, Fig 4).

**Table 8:** Independent and linear dietary and serum fatty acid predictors of targeted clinical indices in bottlenose dolphins.

Clinical Index	Serum Total Fatty Acids	R2	P-value
Cholesterol	C16:1n7	+0.18	0.0009
	C16:1T	+0.36	< 0.0001
	C18:1n7	+0.21	0.0003
	C20:2n6	+0.15	0.002
	C20:5n3	+0.26	< 0.0001
	C22:6n3	+0.44	< 0.0001
	Pristanic acid	+0.22	0.0001
	Total omega-3 fatty acids	+0.37	< 0.0001
Insulin	Pristanic acid to phytanic acid ratio	+ 0.23	0.0001
Hemoglobin	C17:0	+0.16	0.0017
	C20:4n6	+0.28	< 0.0001
	Arachidonic Acid: DHA	+0.29	< 0.0001
	C15:0 (erythrocyte levels)	+0.30	< 0.0001

Lowered serum pristanic acid-to-phytanic acid ratio independently predicted lower insulin (Table 8). Lowered erythrocyte levels of total C18:1 DMA and C20:1n9, as well as raised erythrocyte levels of C15:0, C28:0, and C17:1, also predicted lower insulin. Of these, lowered serum pristanic acid to phytanic acid ratio independently predicted a linear decrease in insulin.

### Raised circulating OCFAs were associated with increased hemoglobin

**Fig 5:**
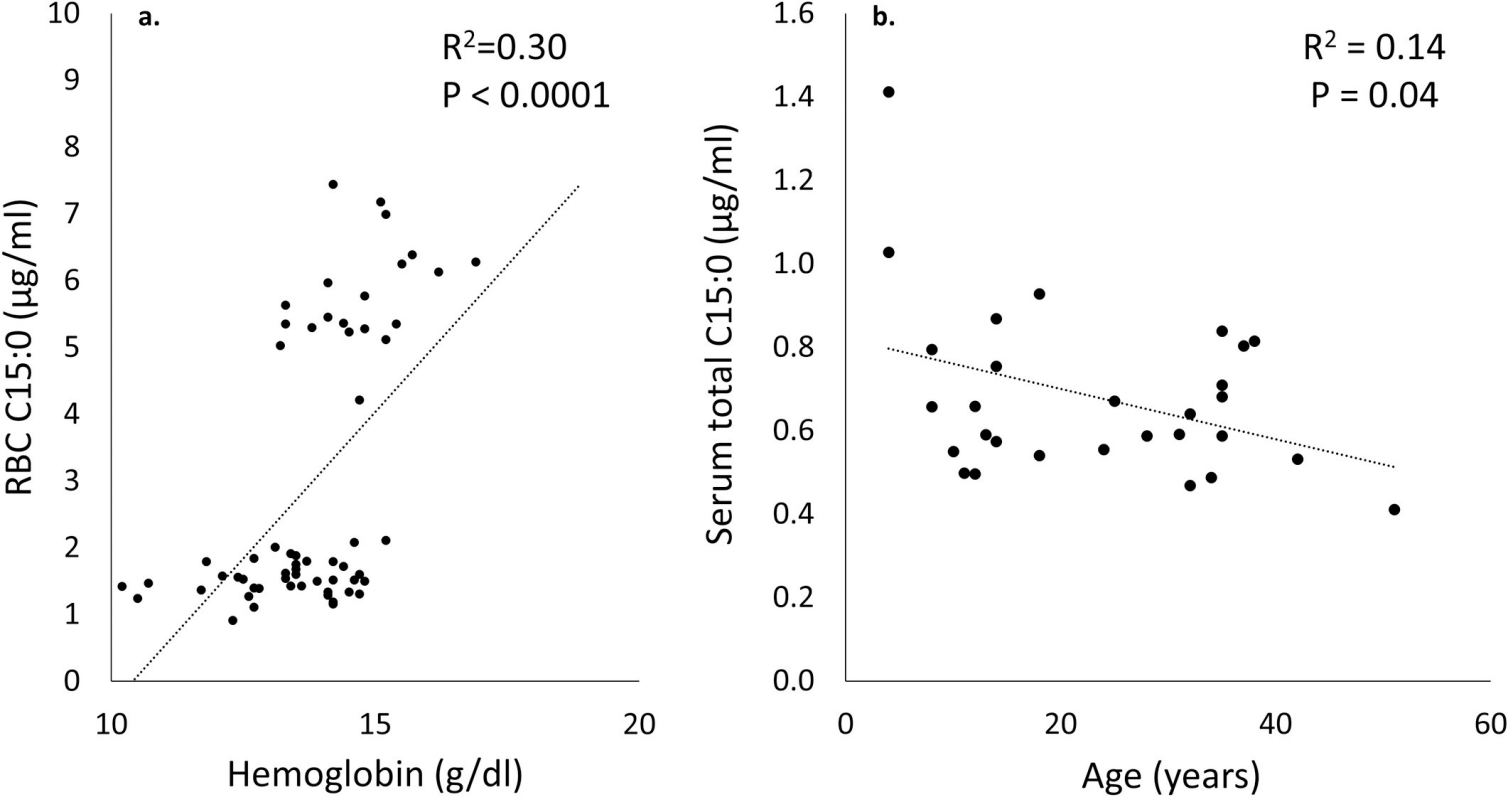
Associations with circulating C15:0, including a) erythrocyte membrane total C15:0 (pentadecanoic acid) as an independent and linear predictor of raised hemoglobin in bottlenose dolphins, and b) inverse associations between serum total C15:0 (pentadecanoic acid) and age in bottlenose dolphins at baseline (Month 0).

Lowered serum C20:5n3, C22:6n3 and raised serum C17:0, C20:4n6, and arachidonic acid to DHA ratio independently predicted raised hemoglobin (Table 7). Lowered erythrocyte total C18:1 DMA and raised erythrocyte C15:0 and C23:0 also predicted raised hemoglobin. Of these, raised serum C17:0, C20:4n6, and arachidonic acid to DHA ratio, as well as raised erythrocyte C15:0 (R^2^ = 0.3, P < 0.0001), independently predicted a linear increase in hemoglobin (Fig 5A). Since increased total C15:0 in erythrocyte membranes independently and linearly predicted raised hemoglobin, and anemia can be an aging-associated comorbidity, we assessed serum C15:0 levels by age among study dolphins at baseline (Month 0); there was a linear, inverse association between age and serum C15:0 levels (R^2^ = 0.14, P = 0.04) (Fig 5B).

At baseline, the 25^th^, 50^th^, and 75^th^ percentiles of hemoglobin in the study population were 12.5, 13.5, and 14.2 g/dl. Here, low hemoglobin (the standard definition of anemia) was defined as hemoglobin < 12.5 g/dl. Given this definition, 7 (23%) animals had anemia and 23 (77%) did not. There were no differences in age or sex when comparing dolphins with or without anemia (26.3 ± 10.3 *versus* 22.3 ± 13.3 years old, P < 0.48; 5/13 (38.5%) versus 2/17 (11.8%) females, P = 0.19) respectively. Independent predictors of hemoglobin at baseline across all 30 dolphins were hematocrit (P < 0.001), mean corpuscular hemoglobin concentration (P < 0.0001), total cholesterol (P = 0.004), LDH (P = 0.016), very low density lipoproteins (P = 0.022), uric acid (P = 0.023), and sodium (P = 0.024). Only hematocrit (P = 0.0007), however, had significant differences when comparing dolphins with or without anemia at baseline.

Independent predictors of hemoglobin among dolphins while on the modified fish diet were hematocrit (P < 0.0001), MCHC (< 0.0001), cholesterol (P = 0.004), alkaline phosphatase (P = 0.038), red blood cell distribution width (P = 0.040), and potassium (P = 0.048). Of these biomarkers, dolphins with anemia were more likely to have lower hematocrit (37.9 ± 2.9 and 46.5 ± 2.6%, P = 0.0002), lower MCHC (30.1 ± 0.3 and 30.9 ± 1.0 g/dl, P = 0.046), higher red blood cell distribution width (16.8 ± 1.9 and 13.8 ± 1.3%, P = 0.003), and lower high-density lipoprotein cholesterol (129 ± 10 and 152 ± 23 mg/dl, P = 0.01) compared to dolphins without anemia.

## Discussion

Bottlenose dolphins, like humans, can develop chronic conditions that progress with advancing age [1–5]. We demonstrate in this prospective interventional study with 30 dolphins that a modified fish diet can attenuate comorbidities of aging, namely anemia, hypercholesterolemia and hyperinsulinemia. These observed clinical benefits were associated with a clear dietary-driven shift in the dolphin serum metabolome, including prioritized-ranked and raised circulating levels of multiple odd-chain saturated fatty acids (OCFAs).

Similar to our pilot study, the current study demonstrated that increased dietary OCFAs successfully increased both serum and erythrocyte total OCFA concentrations in dolphins. In general, an approximate 3.5 to 4-fold increase in daily dietary intake of C15:0 and C17:0 to a mean of 4.5 and 1.1 grams per day, respectively, resulted in similar fold increases of C15:0 and C17:0 concentrations in the serum (to a mean of 28 and 23 μg/ml) and erythrocyte membranes (to a mean of 5.8 and 17 μg/ml), respectively. These data support that circulating OCFA levels can be effectively raised proportionally by the amount of dietary OCFAs ingested. Similar findings have been reported for C15:0 in humans; in contrast, C17:0 blood levels in humans do appear to be higher than expected given dietary C17:0, supporting endogenous production of C17:0 [6, 21–22].

The modified diet raised hemoglobin and hematocrit while lowering red blood cell distribution width and nucleated red blood cells. Among nine dolphins with anemia (≤ 12.5 g/dl) or low-normal hemoglobin (12.6–13.5 g/dl) that were placed on the modified diet, all had normal hemoglobin (>13.5 g/dl) by Month 3. Higher red blood cell distribution width is representative of a greater variety of red blood cell sizes (anisocytosis), and higher nucleated red blood cells reflects active release of new, younger cells into circulation [23]. There were no changes in mean cell volume, mean corpuscular hemoglobin, or iron supporting that the anemia was not due to iron deficiency or other causes for microcytic anemia. There are many potential alternative causes of chronic anemia, including anemia of chronic disease and non-specific anemia of aging. Since we did not find any specific phenotype of anemia at baseline, further studies are needed to better understand etiologies of chronic anemia in dolphins.

Use of global metabolomics enabled a less-biased assessment of over 819 biochemicals detected in these dolphins’ serum 2 hours following a large meal. Here, multiple OCFAs (C15:0, C17:0, and C19:0) were among the highest prioritized metabolites that changed due to the modified diet. Specific dietary driven increases in erythrocyte membrane C15:0 concentrations, as well as serum C17:0 concentrations, independently predicted raised hemoglobin and resolved anemia. Further, at baseline, we report a linear decline in circulating C15:0 concentrations with age in dolphins. This trend suggests that advanced age may negatively impact circulating C15:0 concentrations, and thus, may increase dolphins’ susceptibilities to chronic anemia. Reasons for lower C15:0 concentrations in older dolphins are unknown, and causes including lower food intake with age, should be assessed. In humans, erythrocyte membrane C15:0 concentrations, but not C17:0 concentrations, were significantly lower in premenopausal women with iron deficiency anemia compared to women without anemia [24]. Controlled studies using daily, oral and pure C15:0 and C17:0 treatments (*versus* as part of a complex diet) in accepted animal models are needed to better determine OCFAs’ potential direct role in improving anemia, including aging-driven anemia.

The dolphins’ modified fish diet decreased intake of omega-3 fatty acids, resulting in lower serum and erythrocyte omega-3 fatty acids, including docosahexaenoic acid (DHA) and eicosapentaenoic acid (EPA). In turn, lowered serum DHA and EPA independently predicted lower cholesterol and raised hemoglobin in dolphins. The health benefits of fish oil-based omega-3 fatty acids have been extensively studied, including demonstrated positive impacts on cardiovascular disease, dyslipidemia, and insulin resistance [25,26]. A review of over 11,000 citations, including 61 randomized clinical trials and 37 longitudinal observational studies, however, concluded that omega-3 fatty acids can increase cholesterol [25]. Our current study similarly supports that lower dietary intake of omega-3 fatty acids was associated with lowered serum cholesterol in dolphins. Given dolphins’ high daily intake of omega-3 fatty acids (averaging from 60 to 80 g/day), there are important limitations to comparing dietary omega-3 intake and associated health outcomes between dolphins and humans.

Dietary intake, serum and erythrocyte levels of omega-6 fatty acids, including arachidonic acid (C20:4n6), increased in dolphins on the modified diet. Further, raised serum arachidonic acid concentrations were associated with raised hemoglobin, and raised arachidonic acid-to-DHA ratios were associated with both lower cholesterol and raised hemoglobin. Our previous pilot study with six dolphins on a modified fish diet identified arachidonic acid as an independent predictor of lower insulin [5]. Arachidonic acid participates in proinflammatory pathways, and as such, omega-6 fatty acids have historically been viewed as “bad” polyunsaturated fatty acids that need to be properly balanced with “good” omega-3 polyunsaturated fatty acids to avoid the risk of chronic, inflammatory diseases [27]. Large scale studies in human populations, however, are challenging this argument and demonstrating potential beneficial roles of omega-6 fatty acids, including arachidonic acid, which has both pro-inflammatory and anti-inflammatory properties [28]. A prospective study with 661 older men followed over ten years showed that higher serum omega-6 fatty acids, including arachidonic acid, resulted in a lower risk of developing metabolic syndrome [29]. Pooled analysis from 39,740 adults in 20 prospective cohort studies assessed the role of omega-6 fatty acids in the development of type 2 diabetes and concluded that arachidonic acid was not harmful [30]. These studies have contributed to national dietary recommendations to not exclude omega-6 fatty acids from the diet [31] and are consistent with increased omega-6 fatty acids and associated benefits for dolphins in our current study.

Limitations of our study primarily involved the desire to limit interference with Navy dolphins’ daily activities. While we controlled for no overnight feeding (from approximately 15:00 to 080:0 the next morning) and the size of dolphins’ first morning meal (1/3^rd^ total daily dietary intake), trainers fed 2/3^rd^ of the remaining diet as desired, over a five-hour period (10:00 to 15:00). Additionally, trainers had flexibility in determining the relative amounts of squid and herring that could be fed, limiting the combination of the two to 25% of the total daily diet. Once determined, however, every dolphin was held that individual’s same allocation of herring and squid throughout the study. Because we had a control group and dolphins served as their own controls (to evaluate changes in values during Month 1, 3, and 6 compared to Month 0), this study design limited potential impacts of potential intra-day differences in feeding schedules over a 5-hour period and differences in herring and squid allocations within the 25% allowance between animals that were beyond our controlled feeding regimen.

## Conclusions

In summary, our larger dietary intervention study for Navy dolphins demonstrated that differences in fish types fed to dolphins can influence serum and erythrocyte fatty acid profiles. In turn, changes in circulating concentrations of specific dietary fatty acids, such as increased OCFAs, were associated with observed, improved hematological and cardiometabolic health indices. Future studies, limited to use of pure OCFAs in controlled models, are needed help differentiate these trace fatty acids as causative *versus* associative players in health. This study was limited to 664 known serum metabolites, and future studies including larger numbers of molecules are likely to offer further insights into dietary drivers of cardiometabolic and hematological health in long-lived mammals. Further, additional studies with other dolphin populations under different dietary and management protocols will help verify the relevance of this study’s findings to populations beyond the Navy. Due to shared cardiometabolic and hematologic conditions in long-lived dolphins and humans, the Navy’s dolphin population presents as a unique model for studying dietary lipid-based protective and risk factors, related to chronic conditions, including anemia.

## Supporting information

S1 Data Modified fish diet shifted serum metabolome and alleviated chronic anemia in bottlenose dolphins (Tursiops truncatus): Potential role of odd-chain saturated. (XLSX)
